# Inhibition of Wnt3a/FOXM1/β-Catenin Axis and Activation of GSK3β and Caspases are Critically Involved in Apoptotic Effect of Moracin D in Breast Cancers

**DOI:** 10.3390/ijms19092681

**Published:** 2018-09-10

**Authors:** Sung Min Hwang, Hyo-Jung Lee, Ji Hoon Jung, Deok Yong Sim, Jisung Hwang, Ji Eon Park, Bum Sang Shim, Sung-Hoon Kim

**Affiliations:** College of Korean Medicine, Kyung Hee University, Seoul 02447, Korea; pneuma1@hanmail.net (S.M.H.); hyonice77@naver.com (H.-J.L.); johnsperfume@gmail.com (J.H.J.); simdy0821@naver.com (D.Y.S.); hjsung0103@naver.com (J.H.); wdnk77@naver.com (J.E.P.); eshimbs@khu.ac.kr (B.S.S.)

**Keywords:** breast cancer, Moracin D, apoptosis, FOXM1, β-catenin, GSK3β

## Abstract

Although Moracin D derived from *Morus alba* was known to have anti-inflammatory and antioxidant activities, the underlying antitumor mechanism of Moracin D has not been unveiled thus far. Thus, in the recent study, the apoptotic mechanism of Moracin D was elucidated in breast cancer cells. Herein, Moracin D exerted significant cytotoxicity in MDA-MB-231 and MCF-7 cells. Furthermore, Moracin D increased sub G1 population; cleaved poly (Adenosine diphosphate (ADP-ribose)) polymerase (PARP); activated cysteine aspartyl-specific protease 3 (caspase 3); and attenuated the expression of c-Myc, cyclin D1, B-cell lymphoma 2 (Bcl-2), and X-linked inhibitor of apoptosis protein (XIAP) in MDA-MB231 cells. Of note, Moracin D reduced expression of Forkhead box M1 (FOXM1), β-catenin, Wnt3a, and upregulated glycogen synthase kinase 3 beta (GSK3β) on Tyr216 along with disturbed binding of FOXM1 with β-catenin in MDA-MB-231 cells. Conversely, GSK3β inhibitor SB216763 reversed the apoptotic ability of Moracin D to reduce expression of FOXM1, β-catenin, pro-caspase3, and pro-PARP in MDA-MB-231 cells. Overall, these findings provide novel insight that Moracin D inhibits proliferation and induces apoptosis via suppression of Wnt3a/FOXM1/β-catenin signaling and activation of caspases and GSK3β.

## 1. Introduction

Breast cancer is one of the causes of common tumor deaths in women worldwide [1,2]. Recently, target therapies have been conducted in breast cancer patients. Potent target molecules are considered phosphoinositide 3-kinase (PI3K)/Protein Kinase B (AKT)/mammalian target of rapamycin (mTOR) pathway [3], Notch signaling [4], Her2 [5], and estrogen receptor [6]. 

Emerging evidences reveal that transcription factor Forkhead box M1(FOXM1) is involved in chemoresistance, carcinogenesis, and metastasis [7,8], and so suppression of FOXM1 can be a good strategy for cancer therapy [9]. Certainly, thiostrepton and casticin induced apoptosis and suppressed cell growth in cancer cells by inhibiting FOXM1 [10,11].

Additionally, dysfunction of Wnt/catenin signaling promoted proliferation of mammary and colorectal cancers [12]. In the absence of Wnt ligand, β-catenin is usually degraded by the proteosome system including axin, glycogen synthase kinase 3 beta (GSK3β), and casein kinase 1, while binding of Wnt to a frizzled receptor blocks the activity of destruction complex to degrade β-catenin in the presence of Wnt ligand and so β-catenin is translocated into nucleus [13]. Phosphorylation of GSK3β on Tyr216 induces GSK3β activation and function [14], which is followed by its dephosphorylation on Ser9 [15], though the role of GSK3β is controversial in cancer progression or apoptosis [16]. GSK3β is a primary target of Akt, which inhibits GSK3β function by phosphorylating it on Ser9 in proliferating cells.

For efficient cancer chemoprevention, it is considered one of the potent anticancer strategies to find out effective natural chemicals that selectively induce apoptosis and inhibit proliferation in human breast cancer cells [17,18,19,20].

In the same line, there is accumulating evidence that Moracin D, a 2-arylbenzofuran flavonoid derived from the *Morus alba*, exerts anti-inflammatory, anti-obesity, and antioxidant effects, as *Morus alba* has been traditionally used for diabetes, cough, and heart diseases [21,22,23,24,25] and contains isoprenylated flavonoids, 2-arylbenzopyrans, stilbenes, coumarins, and Diels-Alder adduct compounds [26,27,28].

Nevertheless, the underlying antitumor mechanism of Moracin D was not clearly understood so far. Thus, in the present study, the antitumor mechanism of Moracin D was elucidated in breast cancer cells in association with FOXM1 and β-Catenin/GSK3β signaling with the possibility of a potent pharmaceutical for future agricultural commercialization.

## 2. Results

### 2.1. Cytotoxic Effect of Moracin D in Human Breast Cancer Cells

**Figure 1:**
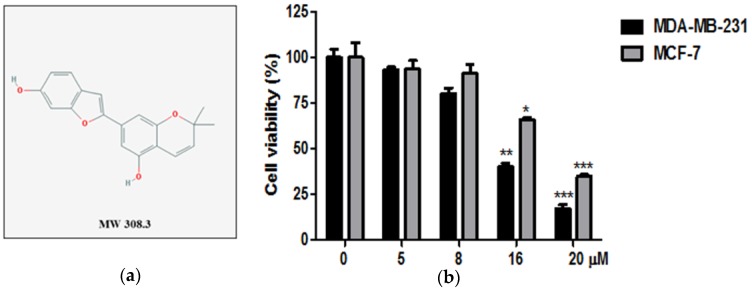
Effect of Moracin D on cytotoxicity in MDA-MB-231 and MCF-7 cells. (**a**) Chemical structure of Moracin D. Molecular weight = 308.3. (**b**) Cells were seeded onto 96-well plates and treated with concentrations of Moracin D (0, 5, 8, 16, 20 μM) for 24 h. Cell viability was evaluated by by 3-[4,5-dimethylthiazol-2-yl]-2,5 diphenyl tetrazolium bromide(MTT) assay. Data represent means ± SD. * *p* < 0.05, ** *p* < 0.01, and *** *p* < 0.001.

The cytotoxicity of Moracin D (Figure 1a) in MDA-MB-231 and MCF-7 cancer cells was evaluated by 3-[4,5-dimethylthiazol-2-yl]-2,5 diphenyl tetrazolium bromide MTT assay. Cells were treated with indicated concentrations of Moracin D (0, 5, 8, 16, 20 μM) for 24 h. Moracin D suppressed the viability in MDA-MB-231 and MCF-7 cells (Figure 1b).

### 2.2. Moracin D Induced Apoptosis in MDA-MB-231 and MCF-7 Human Breast Cancer Cells

**Figure 2:**
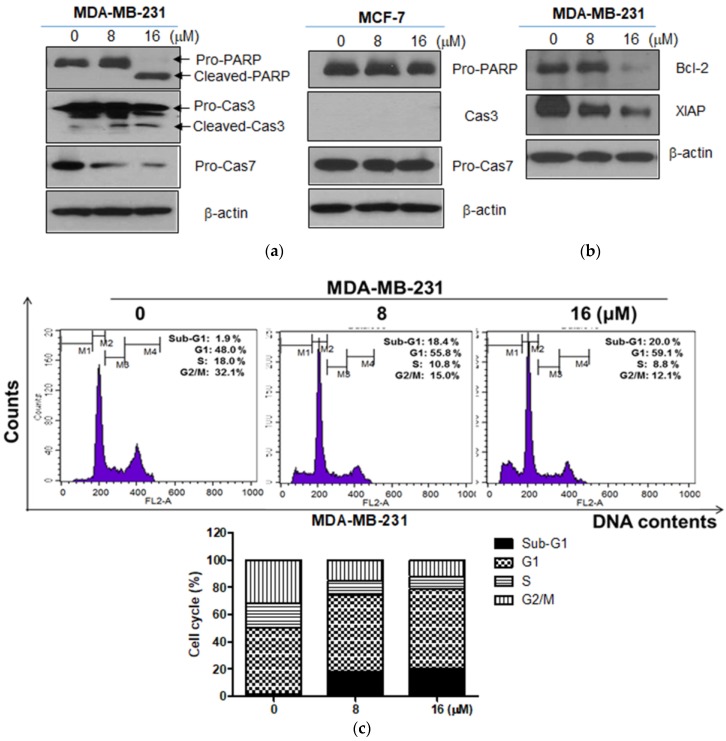
Effect of Moracin D on apoptosis related proteins and apoptosis portion in MDA-MB-231 cells. Human breast cancer cells (MDA-MB-231, MCF-7) were treated with Moracin D for 24 h. (**a**) Cell lysates were prepared and then subjected to Western blotting with antibodies of caspase-3, procaspase-7, and cleaved poly (Adenosine diphosphate ribose (ADP-ribose)) polymerase (PARP). (**b**) MDA-MB-231 cells were treated with Moracin D (0, 8 or 16 μM) for 24 h and subjected to Western blotting for B-cell lymphoma 2 (Bcl-2) and X-linked inhibitor of apoptosis protein (XIAP). (**c**) The stained cells by propidium iodide (PI) were evaluated by fluorescence-activated cell sorting (FACS). The bar graphs show quantification of cell cycle population (%).

To prove the apoptotic effect of Moracin D, cell cycle assay and Western blotting were conducted in MDA-MB-231 cells treated by Moracin D. Moracin D increased the cleavage of PARP and caspase 3 and 7 (Figure 2a). Also, Moracin D attenuated the expression of B-cell lymphoma 2 (Bcl-2) and X-linked inhibitor of apoptosis protein (XIAP) in MDA-MB-231 cells (Figure 2b), while it did not affect the expression of pro-PARP and pro-caspase7 in MCF-7 cells (Figure 2a). However, Moracin D increased sub-G1 accumulation and G1 arrest in MDA-MB-231 cells (Figure 2c). 

### 2.3. Moracin D Effectively Attenuated the Expression of FOXM1 Related Proteins in MDA-MB-231 Cells

**Figure 3:**
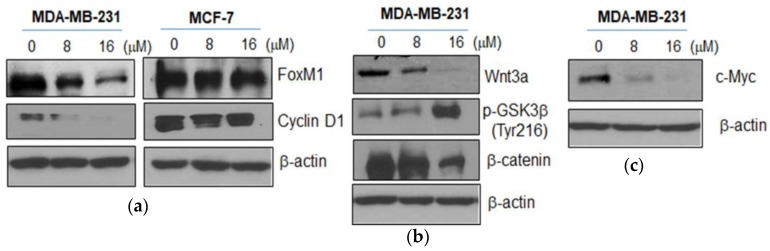
Effect of Moracin D on expression of Forkhead box M1 (FOXM1), Cyclin D1, Wnt3, glycogen synthase kinase 3 beta (GSK3β), β-catenin, and c-Myc in MDA-MB-231 cells. MDA-MB-231 and MCF-7 cells were treated with Moracin D (0, 8, or 16 μM) for 24 h and were subjected to Western blotting with antibodies of FOXM1 and Cyclin D1 (**a**) and also with those of Wnt3, p-GSK3β, β-catenin, and c-Myc (**b,c**).

To confirm whether or not the anticancer effect of Moracin D is related to FOXM1 and Wnt3a/β-catenin signaling, Western blotting was performed in Moracin D treated MDA-MB-231 cells. Moracin D attenuated the expression of FOXM1 and cyclin D1 in MDA-MB-231 cells (Figure 3a), while it did not affect the expression of FOXM1 and cyclin D1 in MCF-7 cells. Likewise, Moracin D effectively suppressed the expression of Wnt3a and β-catenin, enhanced the Tyr 216 phosphorylation of GSK3β (Figure 3b), and attenuated the expression of Wnt target genes, c-Myc in MDA-MB-231 cells (Figure 3c), while it did not affect those proteins in MCF-7 cells (data not shown).

### 2.4. Moracin D Disturbed the Binding between FOXM1 and B-Catenin in MDA-MB-231 Cells

**Figure 4:**
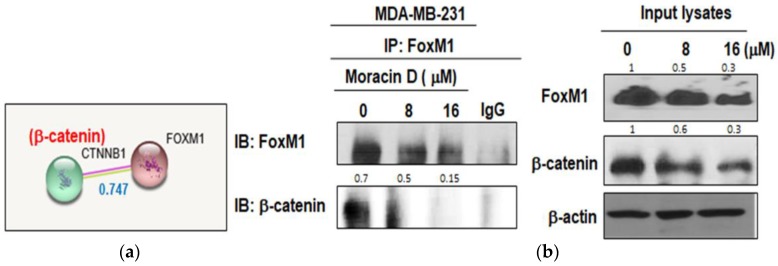
Effect of Moracin D on interaction between FOXM1 and β-catenin in MDA-MB-231 cells. (**a**) STRING database shows interaction score between FOXM1 and β-catenin. Blue number text (interaction score: 0.747). (**b**) MDA-MB-231 cells were treated with Moracin D and Immunoprecipitation (IP) was performed with protein lysates from MDA-MB-231 cells using anti-FOXM1 antibody and then Western blot analysis was performed to detect β-catenin and FOXM1 in input lysates. Input lysates indicate 5% pre-immunoprecipitated samples and β-actin levels confirm equivalent protein loading.

To confirm the inhibitory effect of Moracin D on interaction between FOXM1 and β-catenin, immunoprecipitation was performed in the MDA-MB-231 cell treated by Moracin D. The score of protein–protein interaction (PPI) between FOXM1 and β-catenin was known 0.747 by String database (Figure 4a). As shown in Figure 4b, Moracin D suppressed the binding of FOXM1 and β-catenin in MDA-MB-231 cells in a concentration dependent manner.

### 2.5. GSK3β Inhibitor SB216763 Blocked the Apoptosis Induced by Moracin D in MDA-MB-231 Cells

**Figure 5:**
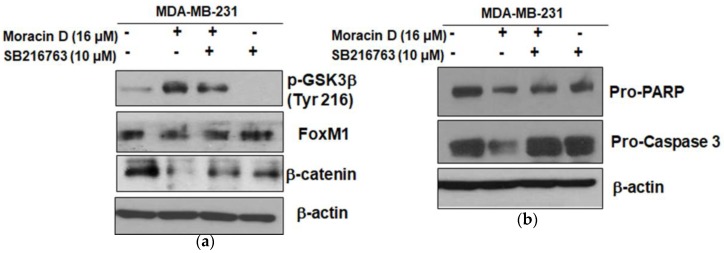
Effect of GSK3β inhibitor SB216763 on pro-PARP, pro-caspase3, FOXM1, and β-catenin in Moracin D treated MDA-MB-231 cells. Cells were treated with Moracin D (16 μM) for 24 h one hour after pre-treatment with 10 μM SB216763 GSK3β inhibitor. Then, Western blotting was performed for FOXM1, β-catenin (**a**), pro-PARP, and pro-caspase3 (**b**).

To explain the downstream role of Wnt signaling in FOXM1-mediated tumorigenesis, GSK3β inhibitor SB216763 was used in MDA-MB-231 cells. Pretreatment of SB216763 blocked the apoptotic effect of Moracin D to inhibit FOXM1, β-catenin, pro-PARP, and pro-caspase3 in treated MDA-MB-231 cells (Figure 5a,b).

## 3. Discussion

In the current study, the antitumor mechanism of Moracin D, a constituent of *Morus alba*, was examined in breast cancer cells in association with FOXM1 and β-Catenin/GSK3β signaling. Herein, Moracin D inhibited the viability of human breast cancer MDA-MB-231 and MCF-7 cells, indicating anticancer potential of Moracin D in human breast cancer cells. Also, Moracin D significantly increased the sub G1 portion and G1 arrest in MDA-MB-231 cells by cell cycle assay, implying the cytotoxicity of Moracin D is mediated by apoptosis in MDA-MB-231 cells. Consistently, Moracin D significantly increased cleavage of PARP and caspase 3 and 7 in MDA-MB-231 cells, but not in MCF-7 cells, demonstrating the caspase dependent apoptosis of Moracin D. Interestingly, caspase 3 was effectively cleaved in Moracin D treated MDA-MB-231 cells, but not in MCF-7 cells, indicating the important role of caspase 3 especially in Moracin D treated MDA-MB-231 cells, not in MCF 7 cells, as caspase-3 is well known to be expressed in MDA-MB-231 cells, but not in caspase 3 deficient MCF-7 cells [29,30,31]. 

FOXM1, which belongs to the Forkhead box (Fox) protein superfamily, is one of the proliferation-associated transcription factors [32,33]. Previous evidence reveals that overexpression of FOXM1 has been implicated in proliferation, metastasis, epithelial-mesenchymal transition (EMT), chemoresistance, and poor prognosis of cancers [8,34,35]. Hence, suppression of FOXM1 is regarded to reduce proliferation and induce apoptosis [36,37]. Previous studies demonstrated that FOXM1 is a key regulator of G1, S, and G2/M progression [38], and so overexpression of FOXM1 has been shown to promote cell cycle progression [39,40]. Also, FOXM1 directly activates transcription of cyclin D1 and cyclin B1, resulting in the improvement of cell cycle progression and cell proliferation [41,42]. Here, Moracin D inhibited the expression of FOXM1 and cyclin D1 in MDA-MB-231 cells, but not in MCF-7 cells, indicating FOXM1 mediated inhibition of cyclin D1. Emerging evidences suggest that FOXM1 upregulates antiapoptotic genes such as Bcl-2 [43] and XIAP [44]. In our study, Moracin D decreased the expression of Bcl-2 and XIAP in MDA-MB-231 cells. 

Wnt/β-catenin signaling plays an important role in cancer progression, including regulation of transformation, cell proliferation, and invasion [45,46]. Wnt3a increases the expression and nuclear translocation of FOXM1, which directly binds to β-catenin for nuclear localization and transcriptional activity [47,48]. Additionally, Yaohui et al reported that GSK3β phosphorylates FOXM1 on serine 474, which induces FOXM1 ubiquitination mediated by FBXW7 [49], while Wnt activation inhibits FOXM1 phosphorylation by GSK3β–Axin complex for deubiquitination and stabilization of FOXM1 [49]. Consistently, Moracin D suppressed the expression of Wnt3*a* and β-catenin, and induced the phosphorylation of GSK3β (Tyr 216) in MDA-MB-231 cells, but not in MCF-7 cells (data not shown), implying that Moracin D inhibits proliferation and induces apoptosis via inhibition of Wnt3a and β-catenin and activation of GSK3β in MDA-MB-231cells. 

Notably, given that MDA-MB-231 cells were more sensitive to Moracin D than in MCF-7 cells via Wnt3a/FOXM1/β-catenin signaling and activation of caspases, different signaling pathways are expected by Moracin D in two breast cancer cells. Hence, we postulate that further study in association with p53 related signaling is required and found in MCF-7 and MDA-MB-231 cells, because MCF-7 cells are p53 wild type cell lines, while MDA-MB-231 cells are p53 mutant cell lines. 

Also, we examined whether or not β-catenin and FOXM1 directly interact in MDA-MB-231 cells. Our results show that Moracin D may affect the binding of FOXM1 and β-catenin in MDA-MB-231 cells, indicating disturbed binding of FOXM1 and β-catenin in Moracin D induced apoptosis. Furthermore, Moracin D attenuated the expression of Wnt target genes such as c-Myc and CyclinD1.

To prove the crucial role of GSK3β in the antitumor effect of Moracin D, GSK3β inhibitor SB216763 was used in MDA-MB-231 cells. Herein, SB216763 blocked the apoptotic effect of Moracin D to repress pro-PARP, pro-caspase3, FOXM1, and β-catenin in MDA-MB-231 cells. 

In summary, Moracin D increased cytotoxicity; sub G1 population; cleaved form PARP; and decreased the expression of pro-caspase 3, Bcl-2, c-Myc, cyclin D1, and XIAP in MDA-MB231 cells. Notably, Moracin D reduced expression of FOXM1, β-catenin, Wnt3a, and upregulated GSK3β on Ty216 partially with disturbed binding of FOXM1 and β-catenin in MDA-MB-231 cells. Conversely, GSK3β inhibitor SB216763 reversed the apoptotic ability of Moracin D to reduce expression of pro-PARP, pro-caspase3, FOXM1, and β-catenin in MDA-MB-231 cells. Taken together, Moracin D inhibits proliferation and induces apoptosis via suppression of Wnt3a/FOXM1/β-catenin signaling and activation of caspase and GSK3β as a potent antitumor pharmaceutical for agricultural commercialization. 

## 4. Materials and Methods

### 4.1. Moracin D Preparation

Moracin D was purchased from Chem Faces Biochemical (Wuhan, China). 

### 4.2. Cell Culture

Human breast cancer MDA-MB-231 (ATCC^®^ HTB-26™) and MCF-7 (ATCC^®^ HTB-22™) cells were purchased from American Type Culture Collection (ATCC). The cells were cultured in RPMI1640 supplemented with 10% fetal bovine serum (FBS) and 1% antibiotic (Welgene, Daegu, Korea).

### 4.3. Cytotoxicity Assay

The cytotoxicity of Moracin D was evaluated using MTT assay. Cells (1 × 10^4^ cells/well) were seeded onto 96-well plate and exposed to various concentrations of Moracin D for 24 h. The cells were incubated with MTT (1 mg/mL) (Sigma Chemical, St. Louis, MO, USA) for 2 h and then exposed to dimethyl sulfoxide (DMSO) for 20 min. Finally, optical density (OD) was measured using a microplate reader (Molecular Devices Co., San Jose, CA, USA) at 570 nm. 

### 4.4. Cell Cycle Analysis

Cells (1 × 10^6^ cells/mL) were treated with Moracin D (0, 8 or 16 μM) for 24 h, washed with phosphate buffered saline (PBS) and fixed in 70% ethanol at −20 °C. The cells were treated with RNase A (10 mg/mL) for 40 min at 37 °C and stained with propidium iodide (50 μg/mL). The stained cell DNA contents were performed in a fluorescence-activated cell sorting (FACS) calibur (Becton Dickinson, Franklin Lakes, NJ, USA) using CellQuest Software.

### 4.5. Western Blotting

Cells (1 × 10^6^ cells/mL) were treated with Moracin D for 24 h, then lyzed in radioimmunoprecipitation assay (RIPA) lysis buffer (with protease inhibitor mixture) on ice, and centrifuged at 14,000× *g* for 20 min at 4 °C. Then, the supernatants were quantified for protein concentration by using protein quantified assay kit (Bio-Rad, Hercules, CA, USA), The proteins lysate samples were separated on 10% Tris gels and transferred to a ECL transfer membrane for detection with antibodies for PARP, caspase 3, caspase 7, FOXM1, Wnt3a, β-catenin, Cyclin D1, c-Myc (Cell signaling Technology, Beverly, MA, USA), phospho-GSK3β (Tyr 216) (Santa Cruz Biotechnologies, Santa Cruz, CA, USA), and β-actin (Sigma, St. Louis, MO, USA).

### 4.6. Co-Immunoprecipitation

MDA-MB-231 cells were lyzed in lysis buffer and then were immuneprecipitated with FOXM1 antibody or normal immunoglobulin G antibody. Protein A/G sepharose beads (Santa Cruz Biotechnology, Santa Cruz, CA, USA) were applied. The last precipitated proteins were subjected to immunoblotting with the indicated antibodies.

### 4.7. Statistical Analysis

For statistical analysis of the data, Sigmaplot version 12 software (Systat Software Inc., San jose, CA, USA) was used. Student’s *t*-test was used for comparison of two groups. The statistically significant difference was set at *p* values of <0.05 between control and Moracin-D treated groups. All data were expressed as means ± standard deviation (SD).

## Abbreviations

FOXM1	Forkhead box
PARP	Poly (ADP-ribose) polymerase
Caspase	Cysteine aspartyl-specific protease
Bcl-2	B-cell lymphoma 2
XIAP	X-linked inhibitor of apoptosis protein
GSK 3β	Glycogen synthase kinase 3 beta
